# Prospective selective embedding of radical prostatectomy specimens is not inferior to full embedding regarding established and new prognostic parameters

**DOI:** 10.1007/s00428-024-03931-4

**Published:** 2024-10-01

**Authors:** Marit Bernhardt, Oliver Hommerding, Tobias Kreft, Leonie Weinhold, Matthias Schmid, Glen Kristiansen

**Affiliations:** 1 Institute of Pathology, University Hospital Bonn, Venusberg-Campus 1, 53127 Bonn, Germany; 2 Institute for Medical Biometry, Informatics and Epidemiology, University Hospital Bonn, Bonn, Germany

**Keywords:** Prostate cancer, Macroscopy, Partial embedding, Complete embedding

## Abstract

The histopathological examination of radical prostatectomy specimens is essential for assessing critical tumor characteristics, including stage, grade, and margins, all of which impact patient prognosis. However, the extent of embedding the prostate has long been a subject of debate, with some advocating partial/selective embedding and others favoring complete embedding. This study establishes a standardized and time-efficient protocol for processing radical prostatectomy specimens with limited embedding while maintaining diagnostic accuracy. Two hundred twenty-six prostatectomy specimens were analyzed, and the results of a highly standardized selective embedding protocol, systematically embedding the apex, the base, the transition to the seminal vesicles, and selected horizontal sections, were compared with full embedding as the gold standard. Non-inferiority testing was conducted by one-sided binomial tests and Pearson-Clopper confidence intervals. Selective embedding provided consistent and accurate diagnostic information with up to 90–98% concordance in pT, margins, ISUP-grade groups, and presence of IDC-P and cribriform tumor growth. In summary, this study establishes an economical standardized protocol for selective embedding of radical prostatectomy specimens with only minimal loss of information.

**Supplementary Information:**

The online version contains supplementary material available at 10.1007/s00428-024-03931-4.

## Introduction

Prostate cancer ranks among the most common types of cancer in men and is one of the leading causes of cancer-related death in men. After a significant increase in incidence due to establishment of prostate-specific antigen (PSA)-based approaches in the late 1990s and early 2000s, these numbers have stabilized in recent years [1–4]. Established risk factors for prostate cancer are age, family history, genetics, and ancestry/race [5]. As prostate cancer primarily affects the elderly, with only rare cases before the age of 40, and an aging population, the number of prostate cancer diagnoses is estimated to increase in the future [1, 2, 6]. Despite significantly improved imaging modalities in recent years, an increase in the workload of pathologists is to be expected [7]. Histopathological examination of radical prostatectomy specimens aims to assess prognostically relevant tumor characteristics. Out of these, tumor stage and tumor grade are the most important. It has to be mentioned that extraprostatic extension in itself is an independent risk factor for cancer recurrence after radical prostatectomy [5]. In addition, positive resection margins are a risk factor for biochemical recurrence after radical prostatectomy though its impact on cancer related mortality remains controversial [5, 8]. Apart from the described tumor characteristics, histomorphological parameters shown to be associated with adverse prognosis are presence of cribriform growth, classified as pattern 4 in the architecture-based Gleason grading system used for prostate cancer, as well as intraductal carcinoma of the prostate (IDC-P) [9, 10].

The International Society of Urological Pathology (ISUP) Consensus Conference on Handling and Staging of Radical Prostatectomy Specimens (Boston, 2009) considered partial embedding and complete embedding to be equally applicable methods for prostate cancer diagnosis, depending on the resources of the respective laboratory. It was noted that partial embedding should be performed according to a fixed protocol and the details of this protocol should be documented in the report [11]. In line with the ISUP recommendation, current national German guidelines for the diagnosis and treatment of prostate cancer recommend total embedding for specimens weighing up to 50 g and offer the option for selective embedding of larger specimens, emphasizing multifocality of prostate cancer and prognostic impact of margin involvement [2]. The European guidelines recommend total embedding, but acknowledge that partial embedding may be considered in prostates > 60 g for reasons of cost-effectiveness [12].

In the past 30 years, multiple studies have been published that employ different approaches and examine variations in inclusion criteria and parameters when dealing with the grossing and embedding of radical prostatectomy specimens [13–24]. Some authors concluded that partial embedding is not inferior to total embedding, while others strongly argued against it. Comparability is limited, as some studies primarily focused on locally advanced tumors, while others compared different cohorts of partially and fully embedded samples [13–15, 17, 18]. When intra-sample comparability was a priority, embedding methods were frequently implemented through step-wise processes, which could potentially lead to increased turnaround times in daily practice or the need to follow complex protocols [16, 21, 23].

The objective of this study was to establish a highly standardized protocol with a limited and preferably identical number of blocks. This approach aimed to reduce processing time while maintaining consistent results for both traditionally but also more recently described prognostical relevant tumor parameters (such as pT, R/margins, grade, IDC-P, and cribriform growth).

## Material and methods

### Specimens

Two hundred twenty-six radical prostatectomy specimens that were processed in our department between April 2022 and February 2023 were included in this study (see Supplement 1). All samples were reported as routine pathology specimens using a standardized synoptic reporting scheme provided by the International Collaboration on Cancer Reporting (ICCR) [25, 26]. Tumor grading, evaluation of resection margins, and extraprostatic and seminal vesicle invasion were performed according to ISUP guidelines [27, 28]. Histological results after partial and complete embedding were recorded in separate evaluation sheets and incorporated into a database (see Supplement 2). Parameters analyzed were as follows: upstaging from pT2 to pT3, upgrading from a negative to a positive resection margin or from a positive resection margin without any therapeutic significance to a resection margin with therapeutic significance, changes in the ISUP grade group (GG), and the amount of cribriform growth as well as the presence of IDC-P.

### Grossing

The pathological processing protocol aimed to be as standardized as possible, in order to achieve a constant labeling of blocks and corresponding location of tissue embedded. All embedding followed a standardized protocol, and macroscopically suspicious lesions were not treated differently. Pathology residents carried out specimen processing after sufficient training. Specimen were received in 4% buffered formaldehyde and fixed for a minimum of 24 h. After fixation, resection margins were inked, and specimen weight was determined only after removal of the seminal vesicle. Standard sized cassettes and glass slides were used for histological examination.

**Fig. 1:**
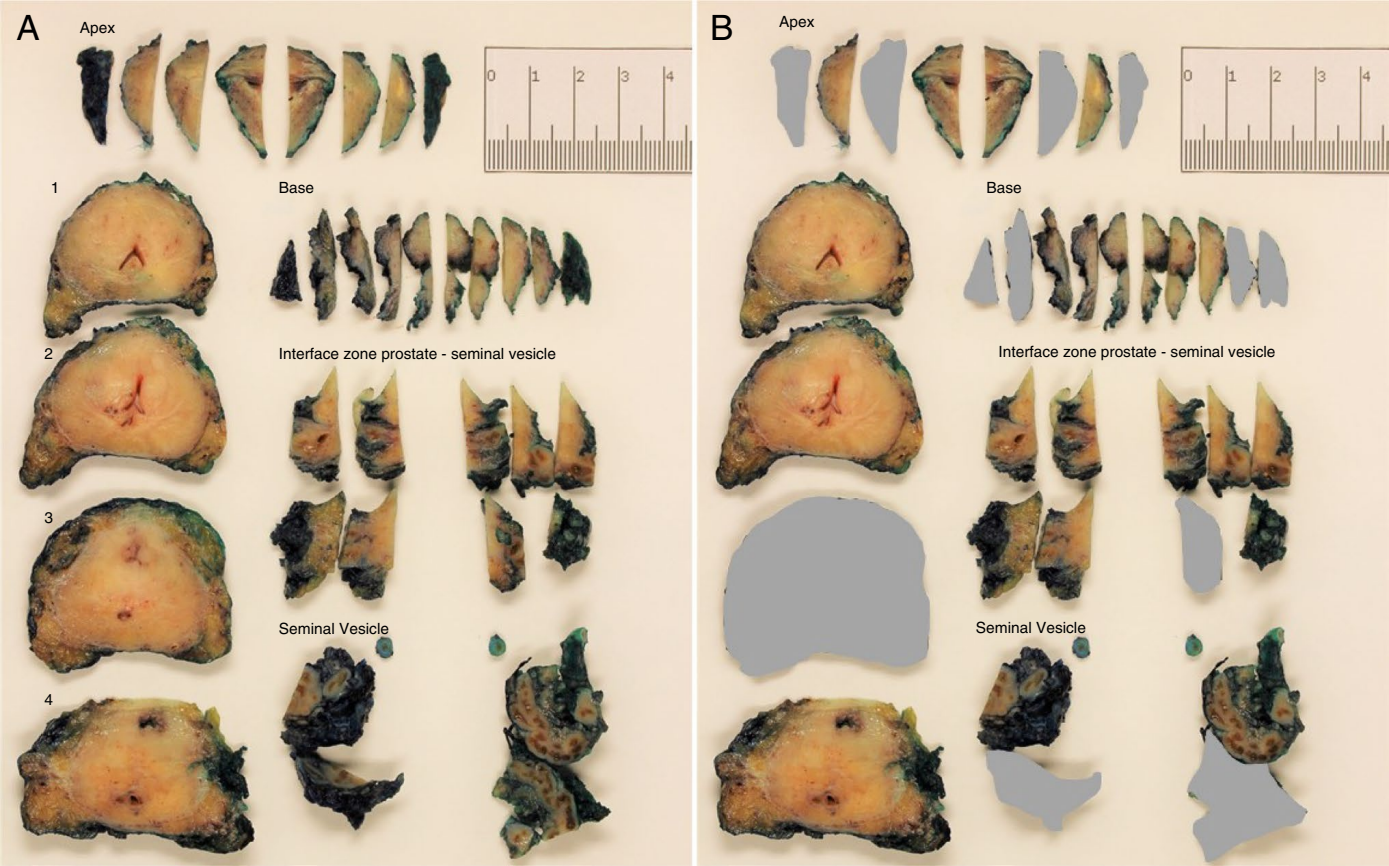
Grossing a prostatectomy specimen. **A** Prostatectomy specimen after sectioning. **B** Parts of the sections chosen for selective embedding are still displayed; meanwhile, ignored parts are covered in grey. Sections chosen are one block each for apical and basal part right and left, two blocks each for interface zone of prostate/seminal vesicle right and left (anatomically peripheral and central zone of the prostate); three central slices, cut in quarters evenly distributed between apical, central, and basal part of the specimen, as well as one block each per seminal vesicle

In order to selectively embed the specimen, the apex and base of the prostate were removed and sliced perpendicularly to the cut surface. One cassette each was taken from left and right side of apex and base. In general, two to three pieces each fit into one cassette, ensuring that the apex, which in general is the most common localization of positive resection margins, was represented sufficiently [29]. Subsequently, the organ was sectioned from apex to base in horizontal slices of 3–4-mm thickness each and numbered in order. All slices were cut in quadrants (ventral right, dorsal right, ventral left, dorsal left). If the slices were too big to fit into four cassettes, slices were cut into six parts, adding a central ventral and a central dorsal part. Likewise, if slices were small, only two cassettes were used. Three horizontal slices per specimen were chosen for selective embedding. Slice selection was aiming at a balanced distribution across the specimen (i.e., slices 1, 4, and 6 for a specimen with a total number of 7 slices) with one slice each from apical, central, and basal portion of the organ (Fig. 1B). Selection of an even distribution was aiming at a representative delineation of the organ and possibly the tumor in order to obtain a realistic impression in terms of tumor morphology and adverse morphologic parameters. In those specimens in which material was harvested for research purposes (biobanking), an additional apical slice was cut in quarters and a frozen section of each quarter was performed. The additional biobank slice was included in the number of blocks for selective embedding. In addition, any tissue that had been investigated in frozen section examination (between two to six blocks) was included in the selective protocol. Tissue of the prostate–seminal vesicle interface was again cut perpendicularly and two cassettes per side were embedded. The increased number of blocks compared to the number selected in apical and basal portion was motivated by the fact that the posterolateral aspect of the organ, represented here, is the most common localization for extraprostatic extension which is an important predictor of prognosis [30]. In addition, seminal vesical infiltration most commonly occurs as direct expansion which, too, can be found in the interface zone of the posterolateral aspect of the organ [31]. Finally, an additional cassette was taken from each seminal vesicle after bisection following ISUP consensus guidelines [31]. Block selection was documented on a standardized scheme used routinely in the department (see Supplement 3). After selective embedding, remaining tissue was processed similarly from apex to base so that no tissue remained (Fig. 1A). All paraffin blocks were cut, mounted on glass slides, and stained with hematoxylin and eosin (H&E).

### Evaluation parameters

**Fig. 2:**
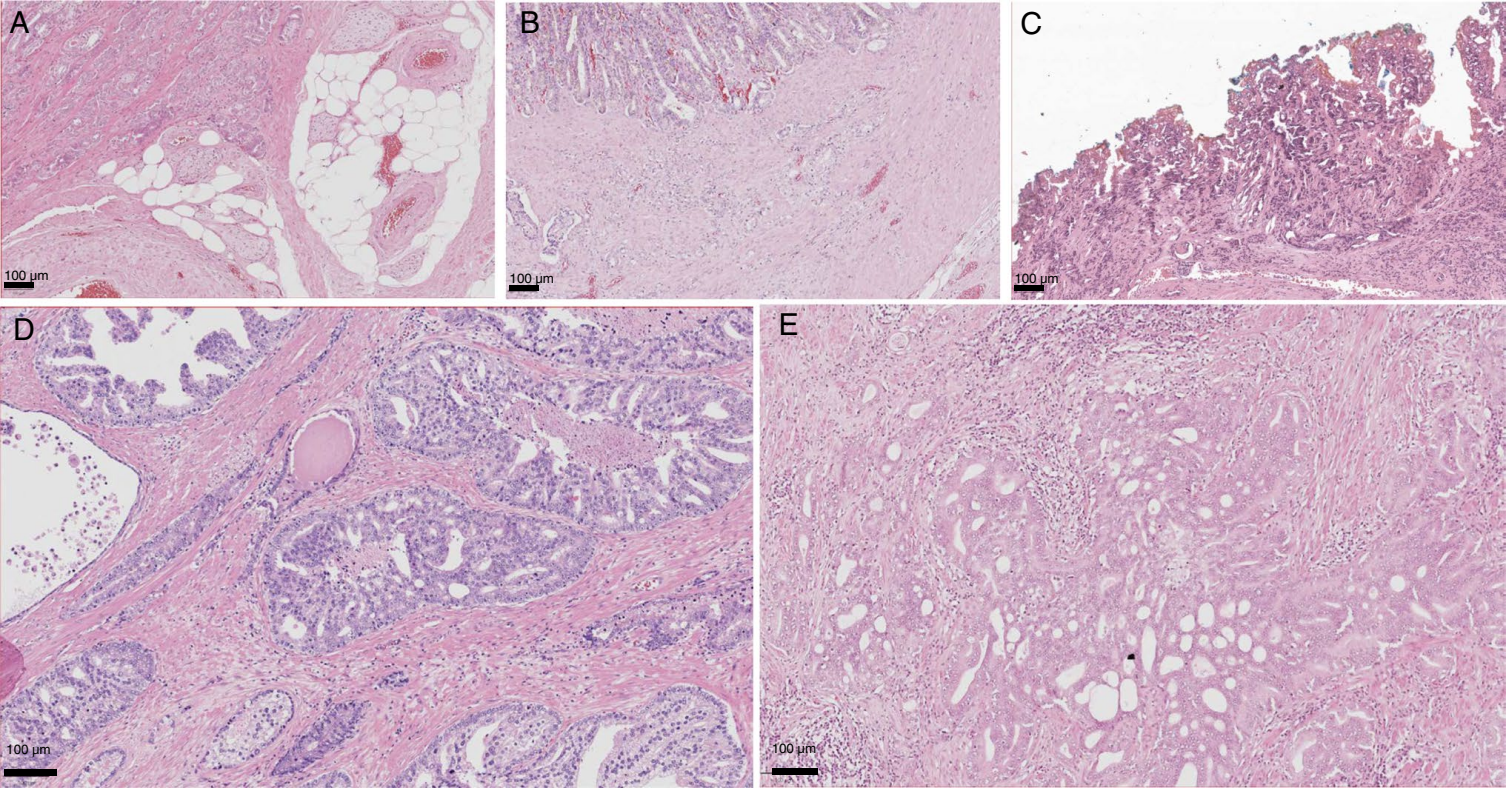
Examples for parameters relevant for staging (**A**–**C**) and histomorphologic predictors of poorer prognosis (**D**, **E**): **A** extraprostatic extension, **B** seminal vesicle infiltration, **C** positive resection margin, **D** intraductal carcinoma of the prostate, **E** cribriform Gleason pattern 4

A positive resection margin was diagnosed if tumor cells were in direct contact with the ink (Fig. 2C), as described previously [29]. Gleason pattern present at the resection margin and cumulative extent of margin involvement (in mm) were recorded separately. Cumulative length of ≥ 3 mm and/or Gleason pattern > 3 at the resection margin were considered as clinically relevant hence significant and a change from insignificant to significant positivity of resection margin was reported separately. This was based on previous findings that Gleason pattern > 3 at the resection margin and length ≥ 3 mm are both independent risk factors for biochemical recurrence after radical prostatectomy [12, 32].

Extraprostatic extension, presence of cribriform Gleason pattern 4, and presence of IDC-P were recorded according to the criteria of the ISUP recommendations [27]. In brief, extraprostatic extension was recorded if tumor cells were found within or adjacent to adipose tissue or perineural spaces of the extraprostatic neurovascular bundle (Fig. 2A). In addition, tumor nodules bulging beyond the gland into the plane of connective tissue as well as tumor infiltration into thick smooth muscle bundles of the bladder neck were regarded as extraprostatic extension, too. Cribriform tumor growth was defined as a continuous sheet of tumor cells forming glandular lumina, visible at 10 × , without the presence of intervening stroma or mucin (Fig. 2E). A change of proportion of Gleason patterns > 5% was considered as a difference between both embedding variations. IDC-P was diagnosed if cancer cells were expanding into and dilating preexisting ducts and acini with preservation of basal cells and tumor cell growth in a trabecular, cribriform, or solid fashion with or without presence of comedonecrosis (Fig. 2D) [27, 30, 33].

### Evaluation

For evaluation, a standardized protocol was established. First, all slides of the selectively embedded blocks were evaluated and results were noted on the protocol (SP2, SP3). Secondly, the remaining slides were analyzed and results were recorded. Final reports were based on a synopsis of the complete slide set. Evaluation was performed by six experienced uropathologists in the department. Ambiguous cases were reassessed by the lead authors (MB, GK). Items recorded in the evaluation form were as follows: patient age, prostate-specific antigen (PSA) levels, number of blocks for selective and complete embedding, maximum ISUP GG and Gleason scores on core biopsy as well as number and location (right/left) of positive cores, Gleason patterns 3, 4, and 5 as well as presence and amount of cribriform Gleason pattern 4 (in % of total tumor volume), presence of extraprostatic extension and seminal vesicle invasion, resection margin and Gleason grade of tumor present at the resection margin.

### Statistics

Statistical analysis was performed using R software version 4.3.0 [34] and IBM SPSS 27 (SPSS Inc, Chicago, IL, USA). Patient characteristics are presented as absolute and relative frequencies for categorical variables and as means with standard deviations (SDs) or median (range) for continuous variables. For group comparisons, cross field tables, Mann–Whitney *U* test, and Spearman correlation were used. One-sided binomial tests and Pearson-Clopper confidence intervals were used to test non-inferiority, i.e., whether the proportion of disagreement of selective embedding and complete embedding (gold standard) was less than 10% for the variables IDC-P, pT, R, presence of cribriform pattern 4 and ISUP grade groups. The significance level was set to 0.05 and *p*-values and confidence intervals were adjusted by the Bonferroni-Holm procedure for multiple testing [35].

## Results

### Change in block numbers

Over 11 months, a total of 226 prostatectomy specimen were collected and fully analyzed according to the study protocol. Resected specimen weighed 41.1 g on average (median 39.9, SD 12.5). Limited processing generated an average of 22.6 blocks (median 23, SD 3.2). In order to fully embed the specimen, on average 19.7 (median 19, SD 6.8) additional blocks were needed. Thus, complete embedding resulted in an 87% increase of blocks. Regarding central slices only, a mean number of 7.1 (median 7, SD 1.7) was obtained, resulting in an average of 10.5 (median 10, SD 3.4) blocks. There was a strong correlation between number of central slices and specimen weight (*p* < 0.001, CC 0.476). On average, blocks of selectively embedded slices represented 47.9% (median 46.3%, SD 12.2%) of blocks obtained in total from central slices (mean 22.7, median 22.5, SD 7.6). Thus, regarding central slices only, total embedding produced a mean increase of 12.2 blocks, equaling an increase of 46.3%. Tumor tissue was identified in all but one selectively embedded specimen (99.56%) and in all fully embedded specimen (100%).

### Change in TNM categories

**Fig. 3:**
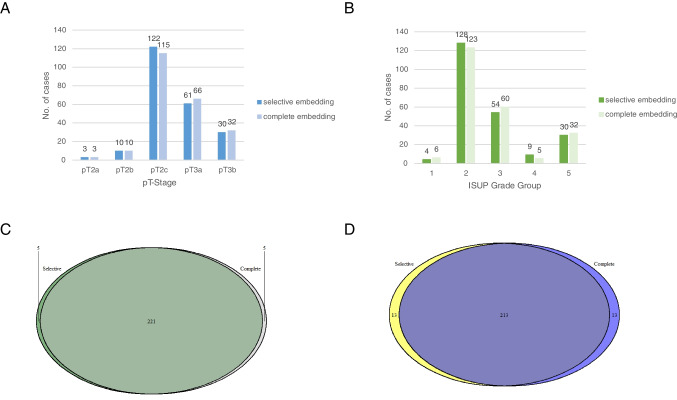
Venn diagrams. **A** Change in pT stage occurred in only a small number of cases with upstaging of pT2c to pT3a/pT3b. **B** ISUP grade groups did not differ significantly between selective and complete embedding; numbers at bars representing absolute numbers and percentage of all cases. The majority of cases did not show different results regarding extraprostatic extension which was detected only after complete embedding in five RP specimens; meanwhile, the same status could be obtained for both selective and complete embedding (**C**) or resection margin where a different resection status between selective and complete embedding was found in 13 RP specimens (**D**)

Complete embedding provided new information resulting in change of TNM categories in 19 cases (8.4%). Out of these change of the pT category was present in seven cases (3.1%) (Fig. 3A). Upstaging of an initial pT2c tumor to a pT3a tumor was shown for five patients (2.2%) (Fig. 3C), to a pT3b in one case (0.4%), and from an initial pT3a to a pT3b in one case (0.4%). Tumors were mostly of intermediate grade (ISUP GG2: four cases, 1.6%; GG3 two cases, 0.8%) with only one case affecting a high-grade tumor (GG5, 0.4%). Complete embedding did not lead to a change in grade group for these cases.

A change of the residual tumor status (R) was present in 13 cases (5.8%) (Fig. 3D). Regarding the 13 margin-positive cases, eight were pT3 (3.5%) and five were pT2 (2.2%). Eight (3.5%) had positive resection margins of clinically significant type, as described above. Change from insignificant to significant positivity in cases with margin positivity already on selective embedding was present in seven cases (3.1%); however, only four (1.8%) of these were classified as margin-positive in the final report due to re-resection tissue sent together with the specimen.

### Change in histomorphological tumor characteristics

A change of amounts of Gleason pattern was called if the percentages differed > 5%. This was seen in 64 cases (28.3%). Changes in Gleason patterns most commonly affected patterns 3 and 4, with pattern 5 only being affected in three cases (1.3%). Regarding the amount of cribriform Gleason pattern 4, a difference > 5% was observed in 28 cases (12.4%). Out of these, 19 affected ISUP grade groups 2 or 3 (8.4%). Presence of cribriform growth was missed by selective embedding in six cases (2.7%).

A difference in amounts of Gleason patterns led to a change in ISUP Grade groups in 22 cases (9.7%) after complete embedding (Fig. 3B). Out of these 22 cases, 12 (5.3%) were upgraded into a higher, and 10 (4.4%) were downgraded into a lower grade group by complete embedding.

Selective embedding missed IDC-P in five cases (2.2%). However, this did not lead to a change in ISUP GG. Two out of the five cases described were GG 2; meanwhile, three were GG 3.

An overview over the changes described is given in Table 1.

**Table 1:** Number of cases per category for both selective and complete embedding

Category	Selective, *N* = 226	Complete, *N* = 226	*p* (chi square)
pT			0.220
2a	3	3	
2b	10	10	
2c	122	115	
3a	61	66	
3b	30	32	
R			0.157
0	171	158	
1	55	68	
ISUP			0.220
1	4	6	
2	128	123	
3	54	60	
4	9	5	
5	30	32	
IDC-P			0.157
Absent	186	181	
Present	40	45	
Cribriform growth			0.157
Absent	109	103	
Present	117	123	

### Correlation of changes in tumor characteristics and number of central slices

The majority of the non-embedded tissue was derived from the central slices. An additional correlation analysis demonstrated that there was no correlation between number of slices/blocks obtained from central slices and change of the parameters pT (*p* = 0.224, CC 0.082), R (*p* = 0.166, CC 0.093), ISUP GG (*p* = 0.460, CC 0.050), and IDC-P (*p* = 0.254, CC 0.077). However, detection of cribriform pattern 4 was correlated with a higher number of blocks/slices (*p* = 0.008, CC 0.177). An overview of the results can be found in Supplement 4.

### Statistical testing for non-inferiority

Upon statistical evaluation for non-inferiority with the gold standard (complete embedding), partial embedding was not inferior regarding the tumor stage (pT, *p* < 0.001) and residual tumor/margins (R, *p* = 0.033) as well as detection of IDC-P (*p* < 0.001) and cribriform pattern 4 (*p* < 0.001) (Table 2). Non-inferiority could not be shown for change in ISUP GG (*p* = 0.503). An additional power analysis showed that the given sample size was sufficient to show the non-inferiority of five variables with at least 80% power, assuming an agreement of at least 95% (alpha = 0.05, Bonferroni-Holm adjusted *p* values) of selective embedding compared to complete embedding.

**Table 2:** Proportion of disagreement of selective embedding and complete embedding. Non-inferiority tests with 10% margin with Bonferroni-Holm adjusted confidence intervals and *p* values

	Proportion of disagreement (%)	95% confidence interval	*p* value
pT	3.1	1.2–6.6	< 0.001
R	5.8	3.1–9.6	0.033
IDC-P	2.2	0.6–5.7	< 0.001
Cribriform*	2.6	0.8–6.2	< 0.001
ISUP	9.7	6.7–13.6	0.503

*Cribriform = cribriform Gleason pattern 4

## Discussion

Several retrospective and only few prospective studies have analyzed loss of information in partially embedded radical prostatectomy specimen with somewhat contradictory results. Hence, there does not seem to be a greater need for yet another study employing a different protocol proving non-inferiority of a—slightly different—selective embedding protocol. The rationale behind this study is as follows: most articles on selective embedding protocols have been published in the early 2000s, so TNM categories did not change in the meantime and the significance of the results obtained is likely to remain valid even 20 years later [36]. However, with the ISUP consensus conferences on grading of prostate cancer in 2005, 2014, and 2019, several new aspects have been introduced that had not been considered in older studies [27, 37, 38]. These are most importantly the introduction of ISUP grade groups in 2014 and the reporting of presence of intraductal carcinoma and invasive cribriform carcinoma/cribriform Gleason grade 4 in 2019 due to their negative prognostic impact [27, 37]. A further aspect addressed, which only became of importance in recent years, is digitization and thereby optimization of workflows by standardization. Here, we aimed at a simple solution to keep block numbers low and comparable at the same time, so that manual adjustments of, i.e., labeling could be kept low.

In radical prostatectomy specimens, stage and grade have been shown to have the highest prognostic impact [5]. Previous studies that employed alternate slice embedding provided highly concordant results in terms of pT stage and involvement of resection margin [19, 20, 24]. Using our method of limited embedding, 94.3% of resection margins were classified correctly and 96.9% of tumors were assigned the correct pT stage. Given the comparatively large number of cases in our study, our method produced a result that was not inferior to the gold standard. Cases that were upstaged to a higher pT after complete embedding were likely to present with a positive resection margin (four out of seven). However, for three of the aforementioned cases, an additional re-resection had been sent with the specimen, resulting in negative margins in the final report. Regarding the protocol, tumor at the resection margin or in close proximity may represent a scenario in which additional material should be embedded in order to provide the best result for the patient. As for the positive resection margin itself, those cases labeled as margin positive after complete embedding most often showed a combination of both extraprostatic expansion as well as presence of IDC-P (six out of 13 cases), so that a high stage tumor with presence of IDC-P on selective embedding may be another good, yet somewhat weaker indicator prompting subsequent embedding of additional tissue.

As mentioned above, most partial embedding approaches mainly focused on stage with tumor grade being only of secondary importance. However, concordance in Gleason score in as many as 98% (75–98%) of cases has been reported [13, 14, 19, 20]. In 2014, the International Society of Urological Pathology Consensus Conference in Gleason Grading of Prostatic Carcinoma introduced five prognostically distinct grade groups (GG), based on categorized Gleason scores, that were able to predict tumor progression more accurately than the previously used three-tiered D’amico Gleason score groups [37]. In our approach, tissue was selected with the aim of obtaining as representative an image as possible of the entire organ and, in particular, of the tumor. In doing so, we were able to correctly identify the ISUP grade group in 90.3% of all samples. However, statistic testing failed to prove non-inferiority of the method by a narrow margin. Given that apart from Fadul et al. and Collette et al. all previous studies were published before the establishment of ISUP grade groups, and the latter did not include ISUP grade groups in their analysis, this study is, to our knowledge, the first to show results for ISUP grade groups for selective and complete embedding [22, 24].

A particularly challenging aspect in the practice of Gleason grading is the definition of variants of pattern 4 [5, 39] Among these are ill-defined (“poorly formed”), fused, glomeruloid, and cribriform glands. The amount of Gleason pattern 4 in patients with either ISUP GG 2 or ISUP GG 3 tumors is strongly associated with outcome [40]. Herein, presence of cribriform growth has been shown to independently influence the probability of distant metastasis as well as disease-specific death of prostate cancer, even when compared to the other patterns of Gleason 4. Hence, the recognition and reporting of cribriform growth was strongly recommended in the last ISUP consensus meeting and incorporated into the current WHO classification [9, 15, 27]. Presence of cribriform growth was correctly identified in this study, as such in the vast majority of cases and the method of selective embedding was shown to be non-inferior upon statistic testing. A minor limitation in this matter may be the positive correlation of embedded (central) tissue and detection rate. So that one might consider embedding additional tissue in equivocal cases in order to detect even minor foci of cribriform growth.

Another important predictor of a poorer prognosis in prostate cancer is presence of IDC-P. Correct recognition is crucial because its presence in prostatectomy specimen has been associated not only with higher stage disease but also with earlier biochemical recurrence after surgery [10]. In our approach, IDC-P was missed in only 3% of selectively embedded cases when compared with the gold standard. However, this did not reflect changes in the ISUP GG, which stayed the same for all patients. Overall IDC-P was present in roughly 20% of cases, matching the frequency of 15–31% described in the literature [5]. As it was the case with the other “new parameter” of cribriform growth, non-inferiority of selective embedding could be shown statistically for the first time. In conclusion, the protocol of selective embedding presented here is an efficient method with comparable result regarding TNM parameters as well as negative morphologic predictors that have only recently been demonstrated to be of greater significance.

The method presented here aimed to use a standardized number of blocks in order to reduce time for laboratory and pathologist work with only minimal and insignificant loss of information. Processing should be both easy, requiring only a limited skillset, and be highly reproducible so that less time would be needed for, i.e., labeling of cassettes, etc.. The mean reduction of blocks needed was 19.7, equaling a reduction of 46.6%. To our knowledge, our method was one with one of the strongest decrease in blocks still achieving a high diagnostic correlation irrespective of specimen size [11, 18, 21]. Even in comparison to the widely known methods of restricted embedding only every second section, the protocol described here achieves a higher degree of information compression [19, 20, 24].

Reducing the number of blocks is crucial in order to save time for pathology because there is a shortage of skilled workers, including not only pathologists but also technicians. The high degree of standardization in grossing is part of preparations for a fully digital workflow. Embedding and slicing are commonly done by hand rather than automated, and it takes—from our experience—approximately 1 to 2 additional minutes in lab turnover time for every block that must be embedded and every slide that must be cut [41, 42]. Loading and unloading machines for dehydration and staining require working time, even though the process itself is likely automated in most laboratories. Moreover, saving resources is not limited solely to the workforce. Considering that healthcare systems are responsible for approximately 4–5% of a country’s carbon dioxide emissions, attempts to reduce these are welcome. The carbon dioxide emissions resulting from one “normal” H&E slide are estimated at between 0.29 and 0.618 kg CO_2_, depending on the specimen type being examined [43, 44]. These calculations encompass all transportation and electricity consumption required to operate the facility, as well as waste disposal. In simpler terms, each block/slide is equivalent to a 1–3-km car ride, which is probably not clear to every pathologist [45].

In recent years, there has been a continuous increase in per capita spending in the healthcare sector. Furthermore, the healthcare system in Germany is pricier compared to other European Union member states [46]. Using the current German hospital billing system, a fully embedded specimen costs about €400, while a partially embedded one is only approximately €215 [47]. Taking into account the minimal differences in diagnosis, the question arises as to whether the time, cost, and resources required for complete embedding are justified.

The aim of our protocol was not only to establish a method, which provided robust results in terms of tumor parameters, but also to reduce technical and intellectual processing time. Herein, a standardized protocol with consistent labeling of cassettes and slides (i.e., left-sided apex always in cassette “B”) allows preparation of cassettes and glass slides before cut up and laboratory work can be automated easily. The use of mega blocks, a technique often suggested to reduce turnover time, is not well suitable in a digital setting as slide scanners but also autostainers for immunohistochemistry most commonly work with standard sized glass slides rather than large format histology slides [48, 49]. Additionally, in order to maintain a lean workflow, a single format is preferable to ensure that all processes are managed alike both within and between specimens. In regard of general short staffing in pathology laboratories which presumably is going to decrease due to future retirement of current practitioners, methods need to be established that are both time and cost efficient while not harming patients [50, 51]. Our protocol fully meets these requirements. Also, a highly standardized embedding will pave the way to a more standardized reporting of RP specimens and will be instrumental for AI-based reporting protocols.

## Conclusion

Selective embedding using only a limited and largely stable number of blocks provides robust results with a low number of false negatives regarding the pathological parameters extraprostatic extension, seminal vesicle infiltration, and resection margins. In addition, there is strong concordance in grading both with Gleason score as well as cribriform and intraductal prostate cancer. These data recommend selective embedding of radical prostatectomy specimens to save both time and resources.

## Supplementary Information

Below is the link to the electronic supplementary material.

Supplementary file1 (DOCX 13 KB)

Supplementary file2 (PPTX 95 KB)

Supplementary file3 (PPTX 186 KB)

Supplementary file4 (XLSX 11 KB)

## Data Availability

Data may be made available by the authors upon reasonable request.
