# Supplementary material for: Prospective selective embedding of radical prostatectomy specimens is not inferior to full embedding regarding established and new prognostic parameters
Source: Virchows Arch. 2024 Oct 1;486(5):931–40. doi: 10.1007/s00428-024-03931-4 (PMC12095424; doi:10.1007/s00428-024-03931-4)
Supplement: Supplementary file 1 — Supplementary file1 (DOCX 13 KB) [file 428_2024_3931_MOESM1_ESM.docx]

**Supplement 1: Patient characteristics**

| **N = 226** |  |
| --- | --- |
| **Age (years)** | 65.9 (mean) (range 47 - 81) |
| **PSA (ng/ml)** | 10.7 (mean) (range 0.7 - 207) |
|  |  |
| **Specimen weight (g)** | 41.1 (mean) (range 17 - 85) |
|  |  |
| **Blocks selective** | 22.6 (mean) (range 15 - 35) |
| **Blocks additional** | 19.7 (mean) (range 4 - 45) |
| **Blocks total** | 42.3 (mean) (range 22 – 74) |
|  |  |
| **ISUP Biopsy** | 3 (mean) (range 1 - 5) |
| **GG 1** | 33 (total) (14.6 %) |
| **GG 2** | 87 (total) (38.5%) |
| **GG 3** | 32 (total) (14.2%) |
| **GG 4** | 36 (total) (15.9%) |
| **GG 5** | 22 (total) (9.7%) |
| **missing** | 16 (total) (7.1%) |

**Supplemental Legend**

**Figure Supplement 2**

Table used in this study to document the results of full vs. selected embedding.

**Figure Supplement 3**

Schematic drawing of a sectioned prostatectomy specimen to document grossing.
