# Supplementary figures and images for: Prospective selective embedding of radical prostatectomy specimens is not inferior to full embedding regarding established and new prognostic parameters
Source: Virchows Arch. 2024 Oct 1;486(5):931–40. doi: 10.1007/s00428-024-03931-4 (PMC12095424; doi:10.1007/s00428-024-03931-4)

## Slide 1
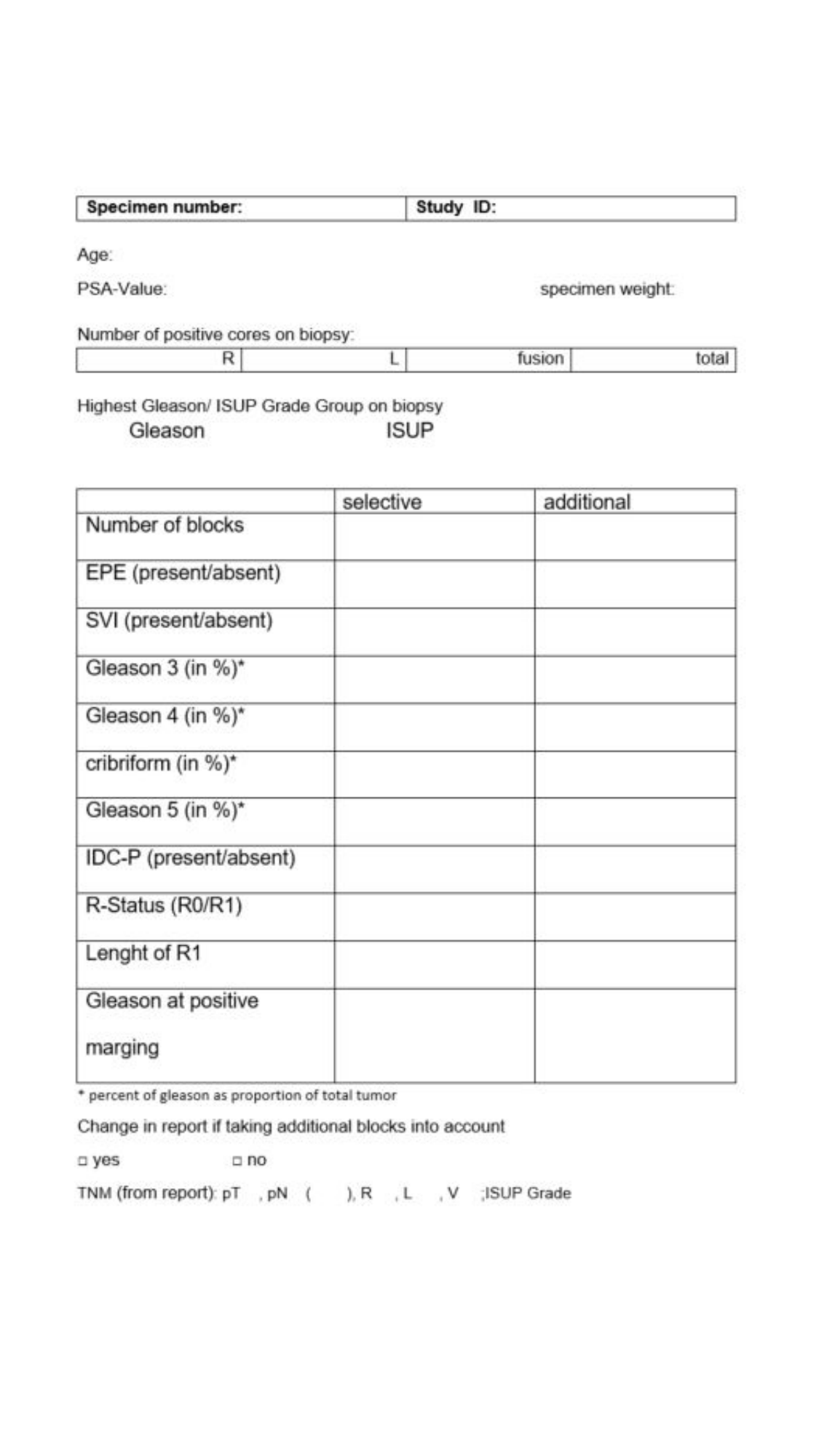

#

Supplement: Supplementary file 2 — Supplementary file2 (PPTX 95 KB) [file 428_2024_3931_MOESM2_ESM.pptx]
