# Supplementary material for: Prospective selective embedding of radical prostatectomy specimens is not inferior to full embedding regarding established and new prognostic parameters
Source: Virchows Arch. 2024 Oct 1;486(5):931–40. doi: 10.1007/s00428-024-03931-4 (PMC12095424; doi:10.1007/s00428-024-03931-4)
Supplement: Supplementary file 3 — Supplementary file3 (PPTX 186 KB) [file 428_2024_3931_MOESM3_ESM.pptx]

## Slide 1
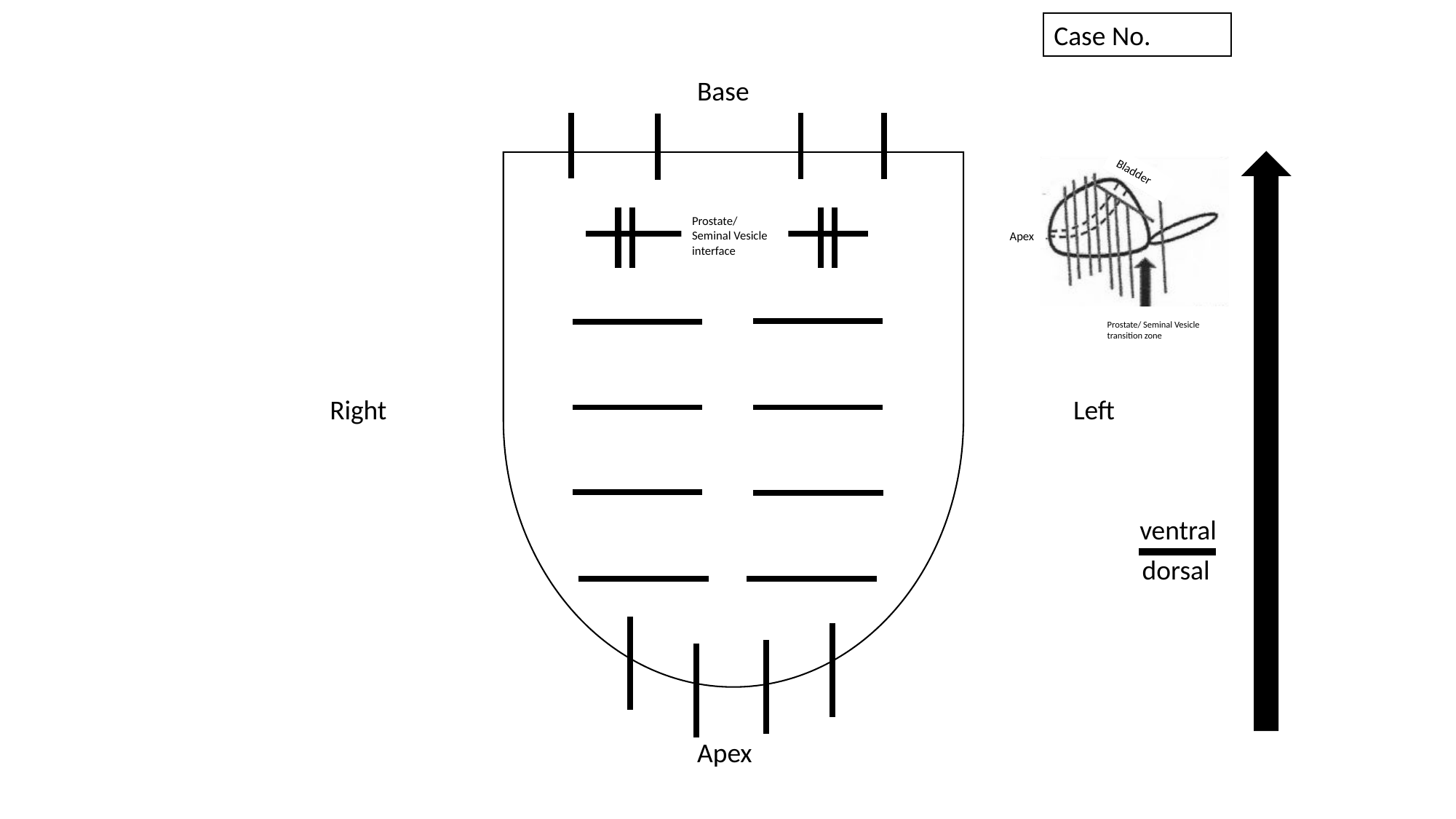

Case No.
Base
Bladder
Prostate/ Seminal Vesicle interface
Apex
Prostate/ Seminal Vesicle transition zone
Right
Left
ventral
dorsal
Apex
